# Tunable Mid IR focusing in InAs based semiconductor Hyperbolic Metamaterial

**DOI:** 10.1038/s41598-017-15493-4

**Published:** 2017-11-10

**Authors:** Mai Desouky, Ahmed M. Mahmoud, Mohamed A. Swillam

**Affiliations:** 1 Department of Physics, School of Sciences and Engineering, The American University in Cairo, Cairo, 11835 Egypt; 2 Electronics and Communications Engineering Department, The American University in Cairo, Cairo, 11835 Egypt

## Abstract

Noble Metals such as Gold and Silver demonstrated for mid IR metamaterials have suffered many obstacles such as: high losses and lack of tunability. The application of doped semiconductors has allowed overcoming the tunability restriction, besides, possessing lower losses as compared to metals. In addition, doped semiconductors have small magnitude of negative real permittivity which is required to realize mid IR Hyperbolic Metamaterials (HMMs). We theoretically demonstrate super focusing based on an all Semiconductor planar HMM using InAs heterostructure. By applying a single slit integrated with doped InAs/InAs HMM, incident light can be coupled to high propagation wave vectors of the HMM modes leading to sub diffraction focusing within the mid IR wave length range. Our proposed structure shows a wide controllable/ tunable operation by changing the doping concentration of InAs. As a consequence, focusing resolution can be tuned over the mid IR ranging from 4.64 μm to 19.57 μm with the maximum achieved resolution is up to 0.045λ at an operating wavelength of 19.57 μm. In addition, we show the effect of substrate refractive index on tuning and enhancing the focusing resolution. Our proposed HMM is an all single based material in which it will not suffer lattice mismatch restrictions during fabrication.

## Introduction

Conventional optical lenses’ performance is known to be constrained by the diffraction limit. This leads to image resolution that is at most about half of the incident wavelength^1^. The diffraction limited imaging is attributed to the fact that evanescent waves holding detailed information about the imaged object cannot propagate in conventional materials. Therefore, they do not contribute to the formation of the final image. In recent years, the metal-dielectric multilayered structure, hyperbolic metamaterial (HMM), has attracted significant interest for its potential to display dielectric property (ε > 0) in two dimensions while exhibiting metallic properties (ε < 0) in the third dimension^2,3^, i.e., hyperbolic dispersion relation. This anisotropy has paved the way for various research topics and applications. HMMs have shown negative refraction of light^4,5^, far field sub wavelength imaging^6^, and sub diffraction focusing^7,8^. Due to their unusual dispersion, HMMs are capable of coupling evanescent waves with high spatial frequency components of free space into propagating modes of HMMs^2,7,8^ and consequently they offer a very promising avenue to surpass the diffraction resolution limit. When covered with a Fresnel zone plate or a diffraction grating that can efficiently excite waves with high wave vectors, subwavelength focusing have been achieved in many HMMs^7–13^. Light focusing has been reported in HMM either by negative refraction of light^10^ or single aperture on HMM^7,8^. These studies have shown focusing from microwave range^7^ to UV range^11^. Focusing in the mid IR range have been reported using metasurfaces or sub-wavelength grating coupler with slight focusing resolution^12–15^. A study has shown that hexagonal naturally accruing hydrogen boron nitride crystal with hyperbolic dispersion momentum space has focusing resolution down to ${\rm{0}}\mathrm{.03}{\rm{\lambda }}$ in the mid IR range^16^.

On the other hand, common metals such as Gold or Silver that are inherently essential ingredients in building HMMs have their natural plasmon resonance in the optical or deep ultra-violet wavelength range. There are no available metals whose plasmon resonance located within the near or mid-infrared (mid-IR) range, which is an extremely important wavelength range for various detection and sensing applications^17^. It should be pointed out here that in order to realize hyperbolic dispersion, the magnitude of the real part of metal permittivity and that of the dielectric need to be of small contrast^4,18^. At mid-IR frequencies, the magnitude of the real permittivity of those metals tends to infinity while there is no dielectric that exhibits such high permittivity value within the same wave length range. This reflects the fact that at these frequencies the electromagnetic properties of noble metals almost resemble those of perfect conductors. This effectively precludes subwavelength metallic structures from supporting localized surface plasmon (LSP) and hence defies any straightforward trials to achieve hyperbolic dispersion in the mid-IR range. Worth mentioning here is that the plasmonic wavelength for a given metal (besides being far away from the mid-IR) is naturally fixed. Hence, having a highly controllable/tunable HMM that is necessary for many applications is not an easy task when using Noble metals^4,19,20^. Thus, it is very important to look into other alternatives to metals that exhibit some controllability and tunability of the plasma wavelength. A perfect candidate for such purpose is moderately or highly doped semiconductors^21^. Many studies have reported the use of either doped semiconductors or graphene for tunable mid IR metamaterials^21–25^. Tuning the Hyperbolic dispersion was studied through inducing field effect in ITO^26^ or by changing the thickness of the dielectric layers in HMM^27^. In this work, we demonstrate an all-semiconductor HMM structure that overcomes the above mentioned challenges and achieve subwavelength focusing in the mid IR range using InAs heterostructures. Doped InAs semiconductor offers the shortest plasmon wavelength with relatively wide tunable plasma wave length^28^. Tuning focusing resolution over the mid IR wavelength range through changing the doping concentration in HMMs has not (to the best of our knowledge) been investigated so far. In this work, we show that the focusing resolution is tunable from 4.64 μm to 19.57 μm by changing the doping concentration. The maximum achieved resolution is up to ${\rm{0}}\mathrm{.045}{\rm{\lambda }}$ at an operating wavelength of 19.57 μm. Finally, we show the effect of substrate refractive index on tuning the focusing resolution. Our proposed doped InAs/InAs HMM can serve as good application for thermal harvesting and/or for subwavelength thermal imaging.

## Results

**Figure 1:**
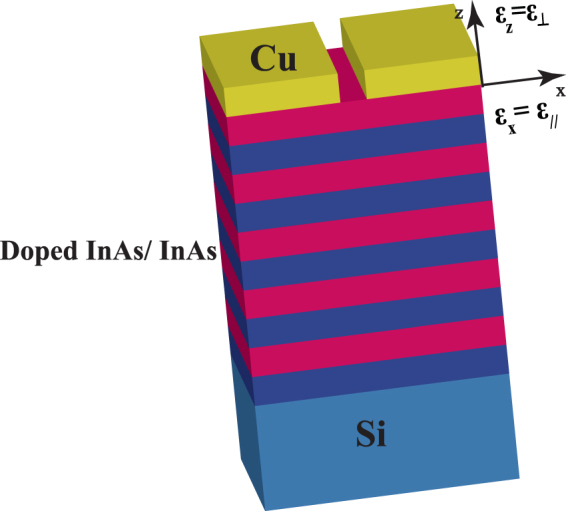
A schematic of our proposed HMM. It consists of 20 alternating layers of doped InAs acting as metal and undoped InAs acting as the dielectric. A Cu layer with 1 μm wide slit is on top of the HMM. The effective permittivities were defined such that ${\varepsilon }_{\parallel }$ is along the x-axis and ${\varepsilon }_{\perp }$ is along the z-axis. ${\varepsilon }_{x}={\varepsilon }_{\parallel } > 0$ and ${\varepsilon }_{z}={\varepsilon }_{\perp } < 0$.

The structure consists of 20 alternating layers of N-doped InAs acting as the ‘metallic’ layer and undoped InAs as the dielectric layer with negative and positive real parts of the permittivity respectively as shown in Fig. 1. Each layer is of thickness 50 nm. A 500 nm Copper layer with 1 μm wide slit was introduced on top of the HMM. The whole structure is supported on Si substrate. Finite difference time domain (FDTD**)** Lumerical has been used for simulating the proposed HMM. A TM polarized Gaussian source was incident from the top of the structure with numerical aperture (NA) of 0.2. The propagation wave vector was defined along the z axis. Perfect matched layer (PML) was defined along the x and z directions.

Since the dimensions are sub-wavelength, effective medium approximation (EMA) can be used to study and predict the dispersion behavior of the proposed HMM^29^. Proper doping concentration was chosen to guarantee negative permittivity in the perpendicular direction. The EMA has been used as follows; Effective permittivity in the perpendicular and the parallel directions are given by ${\varepsilon }_{\perp }={\varepsilon }_{z} < 0$ and ${\varepsilon }_{\parallel }={\varepsilon }_{x} > 0$ respectively.

1 $${\varepsilon }_{\perp }=\frac{{\varepsilon }_{m}{\varepsilon }_{d}}{{f}_{1}{\varepsilon }_{m}+{f}_{2}{\varepsilon }_{d}}$$

2 $${\varepsilon }_{\parallel }={f}_{1}{\varepsilon }_{m}+{f}_{2}{\varepsilon }_{d}$$

Where ${\varepsilon }_{m}$ and ${\varepsilon }_{d}$ are the dielectric permittivities of doped InAs and intrinsic InAs respectively. ${f}_{1}$ and ${f}_{2}$ are the filling ratios of doped InAs and InAs respectively which according to the design dimensions are found to be 0.5 in all simulations. The permittivity of doped InAs ${\varepsilon }_{doped}$ for different doping concentration N_d_ was calculated using Drude model as follows.

3 $${\varepsilon }_{doped}={\varepsilon }_{\infty }-\frac{{\omega }_{p}^{2}}{1+i{\omega }^{2}{\rm{\Gamma }}}$$

4 $${\omega }_{p}^{2}=\frac{{N}_{d}\,.{q}^{2}}{{\varepsilon }_{0}{m}^{\ast }({N}_{{\rm{d}}})}$$

Where ω_p_ is the plasma frequency, Γ is the damping term and m^*^(N_d_) is the effective mass as function of doping concentration. Experimentally obtained values for epitaxial grown doped InAs for five doping concentrations (scattering rate and mobility) were extracted from previous literature^30^. For doped InAs, effective mass is obviously highly dependent on the doping concentration^30,31^. For effective mass calculations, equation (5) is used where ∆E is the band gap calculated by empirical model as follows^31^:

5 $${\rm{\Delta }}E=(\frac{{h}^{2}}{2{m}^{\ast }({N}_{d})}){(\frac{3{N}_{d}}{8\pi })}^{\frac{2}{3}}$$

**Figure 2:**
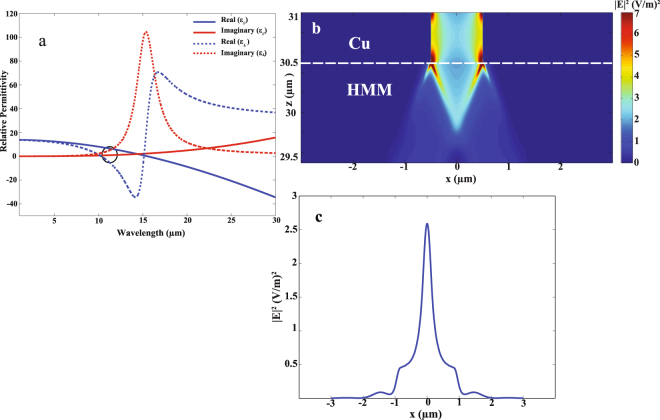
Focusing verification in effective bulk HMM. (**a**) In-plane effective permittivity ${\varepsilon }_{\parallel }$ (red) and out of-plane effective permittivity ${\varepsilon }_{\perp }$ (blue) versus wave length for doped InAs/InAs HMM of N_d_ is 1 × 10^19^ cm^**−**3^. (**b**) Numerical simulation using FDTD shows electric field distribution in XZ plane. Focusing verification in HMM (29.5–30.5 μm). (**c**) Distribution of electric field intensity ${|E|}^{2}$ at ${\rm{\lambda }}$ of 11.56 μm and plane 29.8 μm confirms focusing of incident wave.

For N-doping of N_d_ = 1 × 10^19^ cm^−3^, the plasma wavelength occurs at 10.1 μm. Figure 2a shows the hyperbolic dispersion for type one HMM where ${\varepsilon }_{\perp } < 0$ and ${\varepsilon }_{\parallel } > 0$ for 10.1 μm < λ < 15.3 μm. The wavelength of operation that is marked by black circle in Fig. 2a was chosen (in addition to exhibiting HMM behavior) to have low imaginary parts of the permittivity in both axes to minimize the system losses. FDTD simulation was first conducted for effective bulk structure of HMM using permittivity calculated from EMA to verify the design performance. The operating wavelength was chosen to be 11.56 μm, at which ${\varepsilon }_{\perp }=-8.6+i3.6$, and ${\varepsilon }_{\parallel }=6+i0.5$. Figure 2b shows the electric field distribution ${|E|}^{2}$ in the XZ plane where focusing is clearly observed in the HMM along the z direction. When light propagates in HMM, high spatial K wave vectors can be excited. Diffraction induced by the existent slit results in interference of these high K wave vectors where they propagate along a definite direction. Figure 2c shows the electric field intensity ${|E|}^{2}$ which confirms sub-wavelength focusing of incident light in the effective bulk HMM.

## Numerical simulation and Discussion

In order to confirm focusing, full wave simulation was performed for the entire structure. Doped InAs has tunable plasma wavelength from 4.64 μm to 17.2 μm. Tuning the plasma wavelength will allow focusing at multiple wavelengths over the mid IR range. A layer of perfect electrical conductor with a slit was used instead of Cu which serves as good approximation for metal of very high negative permittivity.

**Figure 3:**
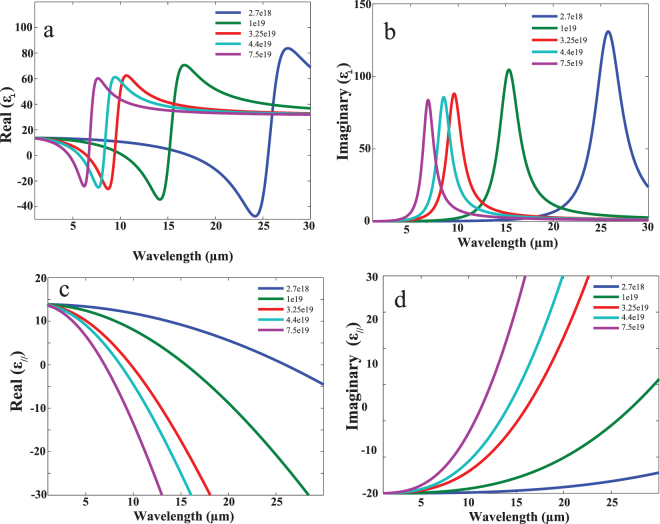
Dispersion relation for doped InAs/InAs HMM. (**a**), Real part, (**b**) Imaginary part of out of plane permittivity ${\varepsilon }_{\perp }$ as functions of the wavelength. (**c**), (**d**) Same as (**a**) and (**b**) but for the in-plane permittivity ${\varepsilon }_{//}$. Five doping concentration N_d_ were studied: 2.7 × 10^18^ (blue), 1 × 10^19^ (green), 3.25 × 10^19^ (red), 4.4 × 10^19^ (turquoise) and 7.5 × 10^19^ (purple) cm^−3^ which yields plasma wave length of 17.2 μm, 10.1 μm, 6.45 μm, 5.7 μm and 4.64 μm respectively.

**Figure 4:**
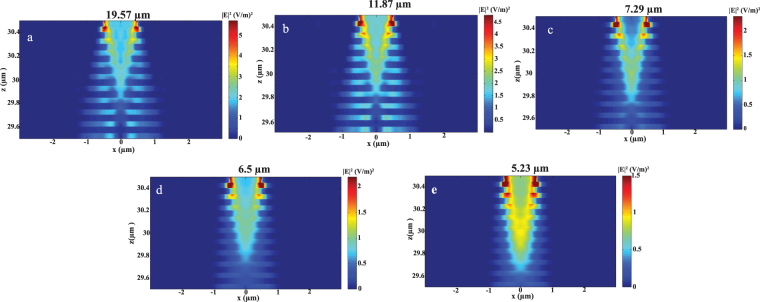
Numerical simulation results of our HMM. It shows the electric field distribution in XZ plane. Focusing verification in HMM structure (29.5–30.5 μm) at wavelengths (**a**)19.57 μm, (**b**) 11.87 μm, (**c**) 7.29 μm, (**d**) 6.5 μm and (**e**) 5.23 μm.

Five doping concentrations were studied as shown in Table 1. It is shown that for larger doping concentrations, the plasma wavelength is blue shifted. Figure 3 shows out of plane ${\varepsilon }_{\perp }$ (a – b) and in-plane ${\varepsilon }_{\parallel }$ (c – d) effective permittivity for different doping concentrations as function of wavelength. It could be observed that for each doping, there is a certain wavelength range that exhibits type one hyperbolic dispersion (where ${\varepsilon }_{\perp } < 0$ and ${\varepsilon }_{\parallel } > 0$). Figure 4 shows the electric field distribution ${|E|}^{2}$ in multilayer doped InAs/InAs HMM at wavelengths 19.57 μm, 11.87 μm, 7.29 μm, 6.5 μm and 5.23 μm for N_d_ of 2.7 × 10^18^, 1 × 10^19^, 3.25 × 10^19^, 4.4 × 10^19^ and 7.5 × 10^19^ cm^−3^ respectively.

**Table 1:** Summary of the focusing resolution for different studied doping concentrations.

N_d_ (cm^−3^)	Plasma wave length (µm)	Focusing Wavelength (µm)	FWHM (µm)	Resolution $(\frac{{\bf{F}}{\bf{W}}{\bf{H}}{\bf{M}}}{{\boldsymbol{\lambda }}})$
2.7 × 10^18^	17.2	19.57	0.88	0.045*λ*
1 × 10^19^	10.1	11.87	0.58	0.049*λ*
3.25 × 10^19^	6.45	7.29	0.78	0.1075*λ*
4.4 × 10^19^	5.7	6.5	0.98	0.1515*λ*
7.5 × 10^19^	4.64	5.23	1.04	0.2*λ*

It can be clearly seen that focusing is achievable for all doping concentrations along defined directions with slight variation in focal length. At wavelength 19.57 µm, the focused field is sharp and confined. For lower wavelengths, the field becomes less sharp where it exhibits the least confinement at the smallest studied wavelength of 5.23 μm. This indicates that as the wavelength becomes smaller with respect to the HMM dimensions, the focusing resolution is degraded. The discontinuity within the electric field in HMM layers can be attributed to the electromagnetic field boundary conditions. The tangential component of the electric field is continuous across each metal/dielectric interface in the parallel direction to HMM layer^2^. In the perpendicular direction, the electric displacement vector is continuous. The electric field will however vary from the metallic to the dielectric layers due to the variation of the permittivity in both layers. Thus, the electric field will experience discontinuity in the perpendicular direction of the HMM to compensate for the change in permittivity. Figure 4b shows the Electric field distribution ${|E|}^{2}$ for N_d_ of 1 × 10^19^ cm^−3^ and at λ of 11.87 μm which confirms focusing down to 0.049λ as previously demonstrated by EMA analysis. The best achievable focusing is 0.045*λ* at 19.57 μm. These values are summarized in table one for the five studied doping concentrations.

As mentioned above, focusing resolution keeps degrading for larger doping concentrations. This can be attributed to the fact that increasing doping concentration enhances the metallic character in the material. Enhancing the excitation of surface plasmon polariton across each metal/dielectric interface increases the dissipation of energy and thus reducing the focusing resolution. This can be clearly seen from the imaginary part of the parallel permittivity in Fig. 3d where damping losses increase for higher doping concentration. Also, the value of the wave length with respect to the slit dimension affects the focusing resolution that we define throughout the manuscript as $\frac{FWHM}{\lambda }$. It is worth mentioning here that, for certain doping concentration, focusing does not occur at just particular wavelength but over range of wavelengths. The wavelength with the best focusing was selected to calculate focusing resolution and depicted in Table 1. To illustrate this, each metal slit generate high K modes due to the effect of diffraction generated at the two slit edges. At certain angle, these high k modes could interfere perfectly at an angle $\tan \,\theta =\frac{\varepsilon //}{\varepsilon \perp }$ with respect to the normal (perpendicular to HMM surface)^7,32^. The direction of propagation is dependent on the choice of the permittivity tensor components at specific wave length that causes the focusing effect. Therefore, perfect interference will be occurring at certain permittivity correspondent to certain wavelength which will have better focusing compared to other wavelengths.

**Figure 5:**
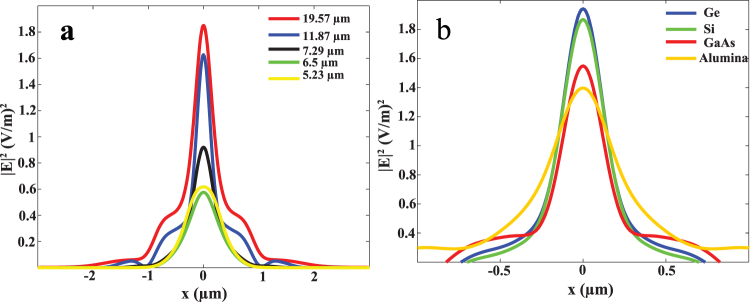
Electric field distribution ${|E|}^{2}$. (**a**) at five wave lengths: 19.57 μm, 11.87 μm, 7.29 μm, 6.5 μm and 5.23 μm, where the resolution is $0.045\lambda$, $0.049\lambda$, $0.1075\lambda$, $0.1515\lambda$ and $0.2\lambda$ respectively. (**b**) ${|E|}^{2}$ for four different substrates: Ge, Si, GaAs and Al_2_O_3_.

Tuning the focusing resolution could be achieved by changing the doping concentration as shown above. Li,G. *et al*. showed that tuning the FWHM could be done by changing the surrounding medium for layered HMM^8^. Here, we study the effect of the substrate refractive index on tuning the focusing resolution. We compared four different substrates Ge, Si, GaAs and Al_2_O_3_ with different refractive indices of 4, 3.42, 3.2, and 1.47 respectively. We choose N_d_ of 1 × 10^19^ cm^−3^ to compare the focusing resolution for the four different substrates. Figure 5b shows that Al_2_O_3_ has the least focusing resolution of $0.0986\lambda$ and the focusing is enhanced by increasing the refractive index of the surrounding medium. The maximum achieved resolution is for the highest index substrate Ge which is $0.0457\lambda$ that is even better than our previously studied HMM supported on Si.

## Conclusion

We theoretically show that sub-diffraction focusing can be achieved in the mid IR range using doped InAs/InAs HMM from 4.64 μm to 19.57 μm. Tuning the operating wavelength and the focusing resolution is achieved by changing the doping concentration. The maximum resolution reached in our study is $0.045\lambda$. We also demonstrate that further tuning of the focusing resolution is achievable upon using high refractive index substrates. Finally, choosing material of lower losses and being tunable opens the way to realize focusing over the mid IR range with extremely high resolution limit. In addition, planar hyperbolic metamaterials are easy to fabricate specially that our proposed HMM design is an all single based material and will be immune to lattice mismatch constrains. Focusing in the mid IR wave length range with sub-wavelength resolution limit is an efficient way to realize thermal harvesting and/or thermal imaging applications.
